# Sinonasal Malignant Melanoma Variant: A Case Report

**DOI:** 10.7759/cureus.27813

**Published:** 2022-08-09

**Authors:** Alshema Alqurashi, Omar Ayad N Alsulami, Mohammad O Albakrei, Rehab Fadag

**Affiliations:** 1 Otolaryngology - Head and Neck Surgery, King Fahad Armed Forces Hospital, Jeddah, SAU; 2 Medical School, King Abdulaziz University, Jeddah, SAU; 3 Otolaryngology - Head and Neck Surgery, King Faisal Specialist Hospital and Research Centre, Jeddah, SAU; 4 Histopathology, King Fahad Armed Forces Hospital, Jeddah, SAU

**Keywords:** head & neck cancer, hemosiderin, melanin, melanoma, blue cell tumour

## Abstract

Sinonasal malignant melanoma (SMM) is a rare malignant tumour among head and neck cancers predominantly found in adults 60 years and above. The commonly reported symptoms for sinonasal tumour lesions are nasal obstruction and recurrent, painless epistaxis as the symptoms are non-specific and can delay the diagnosis. Moreover, melanoma has a poor prognosis regardless of its location. We report an 86-year-old female patient presenting with recurrent, painless epistaxis from the nasal cavity. Anterior rhinoscopic examination revealed a bluish-black, bleeding mass completely obstructing the left nasal nare. Contrast-enhanced computed tomography of the nasal cavity and sinus region showed a polypoidal soft tissue attenuation with heterogeneous enhancement completely filling the left nasal cavity. The patient underwent endoscopic excision. Histopathology of the specimen showed a small, round and blue cell tumour which immunohistochemistry found to be positive for S100 and HMB 45. After surgical resection, the patient received chemotherapy and radiotherapy. Sinonasal malignant melanoma is a rare, aggressive tumour that has a very poor prognosis. Contrast-enhanced computed tomography of the nasal cavity and paranasal sinuses is the imaging modality of choice which reveals the enhancing mass. There is no optimal management strategy for SMM. Surgical resection is the first-line treatment but is limited due to the complex anatomy of the sinonasal region.

## Introduction

Melanocytes are neural crest cell derivatives that are found all over cutaneous and mucosal surfaces [1]. In the nasal cavity, they are located in the mucous membranes, glands, superficial and deep stroma of the septum and turbinates, as well as among the olfactory epithelium's supporting cells [2]. Malignant melanoma is a neoplasm that originates from abnormal melanocytes or neural crest cells [3]. Malignant melanomas of the nasal cavity although rare, are aggressive and have a poor prognosis, irrespective of treatment with a five-year survival rate of 13% to 45% [4]. They represent between 0.5% and 2.5% of all melanomas [5]. Histological findings are used to make a diagnosis, however, as the symptoms are non-specific and develop gradually, thus patients present at a later stage with more advanced disease causing a delay in diagnosis [6, 7]. The mainstay of treatment for sinonasal malignant melanoma (SMM) is complete tumour resection [8]. However, due to limited surgical visibility, the complex anatomy of the sinonasal region, and the involvement of adjacent vital structures, complete tumour excision with clear surgical margins is difficult. Therefore, while postoperative radiotherapy and adjuvant chemotherapy are a part of the treatment protocol, they offer limited therapeutic value [1].

## Case presentation

An 86-year-old female presented to the otorhinolaryngology clinic in August 2021, with a chief complaint of recurrent episodes of bleeding from both nostrils for the past two weeks. The bleeding was not active at the time of presentation. The patient gave a history of hypertension and dyslipidemia. The hypertension was diagnosed eight years ago and she was prescribed perindopril and atenolol while the dyslipidemia was diagnosed four years ago for which she was prescribed atorvastatin. There was no history of trauma or inserted foreign bodies. Anterior rhinoscopy examination revealed a bluish black mass occupying the left nasal cavity and minimal ulcerative changes on the left lateral dorsum of the nose in contrast to the right nasal cavity which was normal on examination. Examination of the ears revealed normal bilateral tympanic membranes and no signs of hearing impairment. In addition, general physical examination was insignificant, and the vital signs were within normal range.

**Figure 1:**
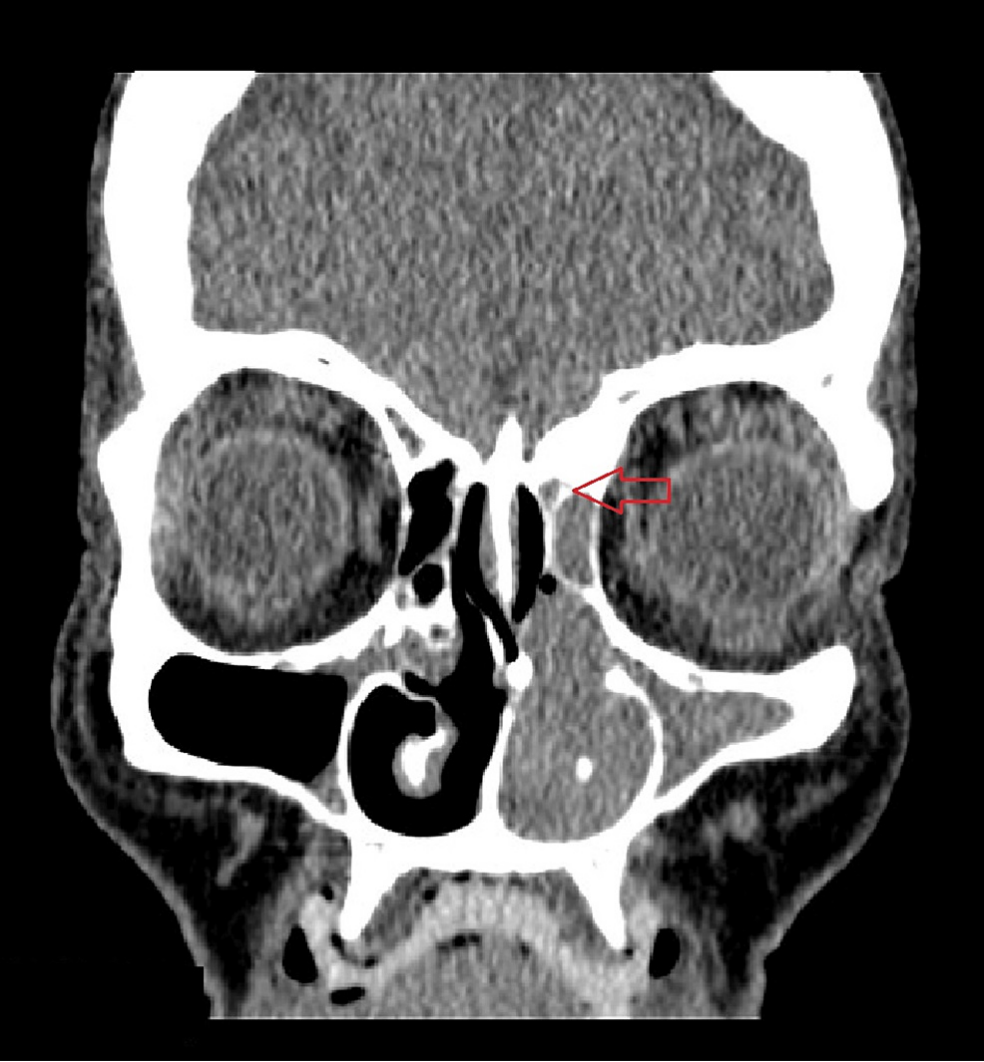
Coronal CT scan showing a mass (red arrow) extending superiorly to the ethmoid sinus

Contrast-enhanced computed tomography (CT) of the nasal cavity and paranasal sinuses showed a heterogenous-enhancing left nasal cavity mass, involving the turbinate as well as obliteration of osteomata complex and extension into posterior nasopharynx causing a mass effect on the medial wall of the maxillary sinus, leading to obstruction associated with diffuse mucosal thickening of the left maxillary sinus with extension superiorly to the ethmoid sinuses. Mucosal thickening was also evident in both ethmoid sinuses and the left frontal sinus (Figure 1). The lamina papyracea and cribriform plate were intact. No adjacent bony erosions or destructions were observed. There were no significantly enlarged cervical lymph nodes.

**Figure 2:**
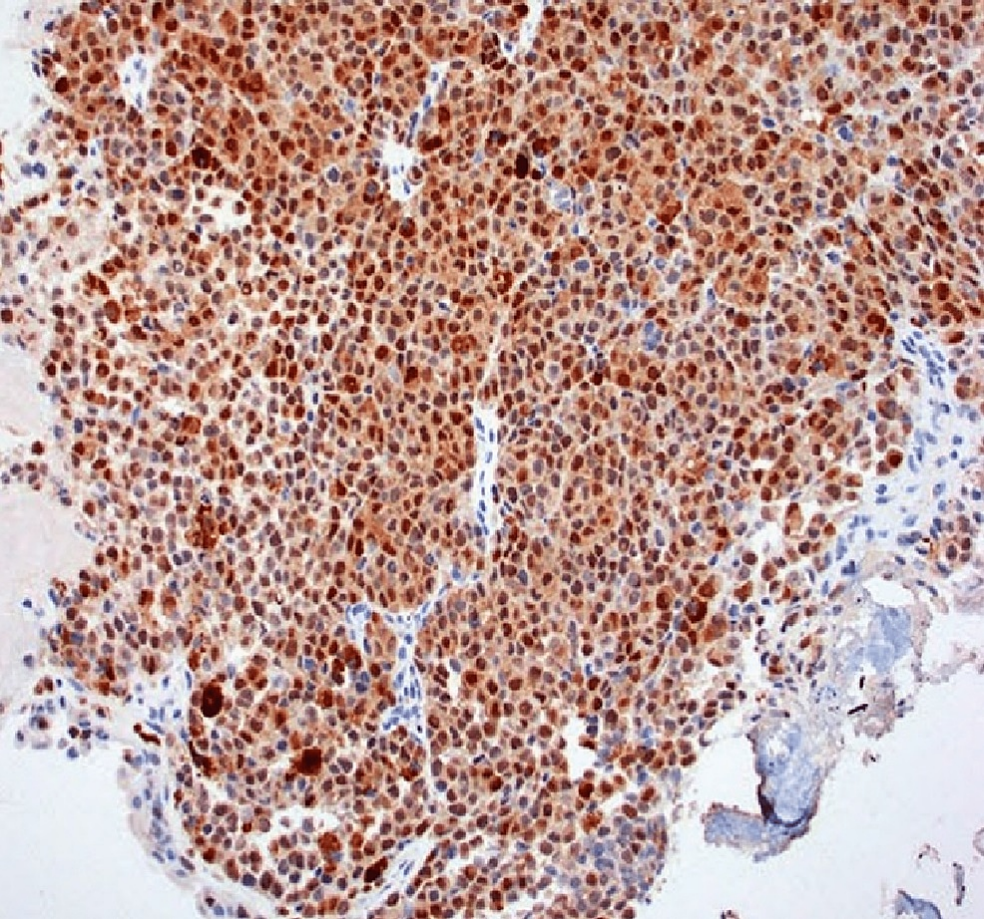
Immunohistochemistry of the biopsy showing malignant melanocytes positive for S100

**Figure 3:**
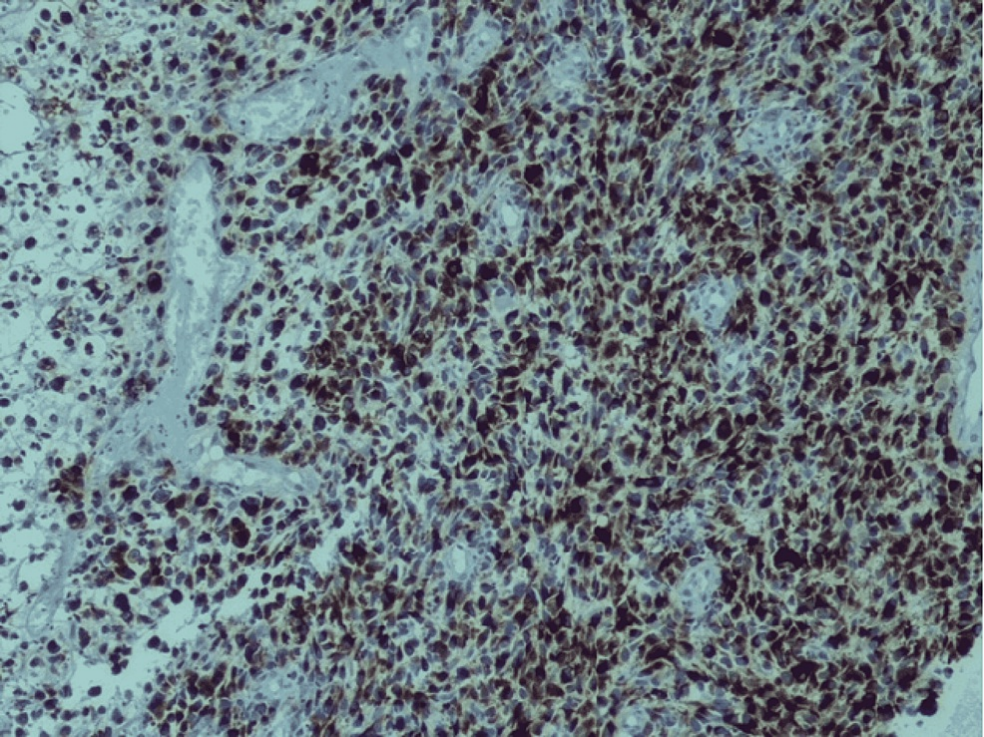
Immunohistochemistry of the biopsy showing malignant melanocytes stain positive for HMB 45

The patient underwent an endoscopic resection under general anaesthesia. Intra-operative findings showed a friable, sessile mass arising from the left lateral wall of the nose. Haemostasis was achieved. The surgery was uneventful and the patient was discharged after two days of hospitalisation. Histopathology of the specimen revealed a malignant tumour composed of sheets of round to oval tumour cells with spindle, pleomorphic, rhabdoid, plasmacytoid and small cell cytoplasm and regular round hyperchromatic nuclei, melanin pigment was seen at places suggestive of small round blue cell tumour which can be considered a variant of sinonasal melanoma. Immunohistochemistry of the biopsy was found to be positive for S100 and HMB 45 (Figures 2-3). Afterwards, the patient was referred to a tertiary centre for initiation of chemotherapy and radiotherapy.

## Discussion

Melanoma of the head and neck are uncommon, accounting for 0.4% to 1.8% of all malignant melanoma. The most common sites for melanomas are the anterior portion of the nasal septum, followed by the lateral wall of the nasal cavity [9]. Sinonasal melanoma is an aggressive and rare lesion with an incidence of 0.2-1 cases per million people [10, 11], making up less than 1% of all melanomas and less than 5% of all sinonasal tract neoplasms. Where there is a high incidence of malignant cutaneous melanoma, the incidence of malignant mucosal melanoma is lower. While sinonasal melanoma can occur at any age, it primarily affects people over the age of 60 and only rarely affects young people [12]. Compared to cutaneous melanomas, the tumour’s survival rates are still poor because of its rarity, anatomical complexity, and unique immunohistochemical and histopathological characteristics. Throughout the literature, the vast majority of patients had either nasal obstruction, epistaxis, or both, or loss of smell [13]. The average delay between the onset of symptoms and diagnosis was 5.6 months [14]. Our case involves an older female patient who experienced recurrent, painless epistaxis of both nostrils. She was eventually found to have metastatic SMM of the maxillary and ethmoid sinuses leading to obstruction associated with diffuse mucosal thickening of the left maxillary sinus with extension superiorly to the ethmoid sinuses. Mucosal thickening was also evident in both the ethmoid sinuses and the left frontal sinus.

To evaluate staging, location, and regional extension as well as to rule out lesions affecting the meninges, brain tissue, and major arterial structures, CT and magnetic resonance imaging are required. Displacement of the periorbita is typically diagnosed on CT, with a reported negative predictive value (NPV) of 86% and a positive predictive value (PPV) of 75% [15]. Computed tomography represents the best modality with which to assess the presence of bony remodelling or bony invasion. In our case, no adjacent bony erosions or destructions were observed. Also, there were no significantly enlarged cervical lymph nodes at diagnosis. A tumour can be resected from the periorbita, but the tumour-invading ocular muscle will typically require orbital exenteration and thus an open approach. Endoscopic orbital exenteration has been described but is not widely practised [16]. In our case review, the patient underwent endoscopic excision. Although studies have shown that patients who underwent endoscopic resection had significantly higher survival rates, an external approach is frequently used in cancers with advanced stages [4, 17]. However, external or combined (endoscopic and external) approaches are still advised as viable surgical choices in SMM extensively invading surrounding and bone structures [18, 19]. Radical surgery and adjuvant radiotherapy and/or chemotherapy for SMM usually carry a poor prognosis with reported five-year survival rates varying across the studies (22% to 80%) [4, 20-22]. In a meta-analysis conducted by Gore et al., 54.6% was the recurrence rate. This large meta-analysis confirmed that there was no survival advantage for combined radiotherapy + surgery or chemoradiotherapy + surgery versus surgery alone [23]. However, postoperative radiotherapy and adjuvant chemotherapy are a part of the treatment protocol, but they offer limited therapeutic value.

Treatment of recurrent epistaxis of unknown origin with packing/cautery should be followed by timely endoscopy with potential biopsy and then imaging. The prognosis and available treatments for sinonasal melanoma are significantly impacted by early diagnosis. Imaging latency could delay diagnosis and therapy and have a negative impact on the course of the disease. We advise primary care doctors to promptly refer patients who experience frequent bouts of epistaxis for additional attention or to order imaging exams for their sinuses.

## Conclusions

Sinonasal malignant melanoma is a rare, aggressive tumour that has a very poor prognosis. Contrast-enhanced computed tomography of the nasal cavity and paranasal sinuses is the imaging modality of choice which reveals an enhancing mass. Surgical resection is the first-line treatment which has its own limitations due to limited surgical visibility and the complex anatomy of the sinonasal region. Surgery in combination with radiotherapy and adjuvant chemotherapy is utilised, however, it does not always improve chances of survival as local recurrence and distant metastasis are common outcomes and are poor prognostic indicators of the disease.

## References

[1] Meng XJ, Ao HF, Huang WT (2014). Impact of different surgical and postoperative adjuvant treatment modalities on survival of sinonasal malignant melanoma. BMC Cancer.

[2] Zak FG, Lawson W (1974). The presence of melanocytes in the nasal cavity. Ann Otol Rhinol Laryngol.

[3] Lamichhane NS, An J, Liu Q, Zhang W (2015). Primary malignant mucosal melanoma of the upper lip: a case report and review of the literature. BMC Res Notes.

[4] Dauer EH, Lewis JE, Rohlinger AL, Weaver AL, Olsen KD (2008). Sinonasal melanoma: a clinicopathologic review of 61 cases. Otolaryngol Head Neck Surg.

[5] Iversen K, Robins RE (1980). Mucosal malignant melanomas. Am J Surg.

[6] Alves IS, Berriel LG, Alves RT, Pinto MB, Oliveira CF, Cazzotto AC, Moura WV (2017). Sinonasal melanoma: a case report and literature review. Case Rep Oncol Med.

[7] Sanderson AR, Gaylis B (2007). Malignant melanoma of the sinonasal mucosa: two case reports and a review. Ear Nose Throat J.

[8] Lund VJ, Stammberger H, Nicolai P (2010). European position paper on endoscopic management of tumours of the nose, paranasal sinuses and skull base. Rhinol Suppl.

[9] Wenig BM (2007). Tumors of the upper respiratory tract, Part A - Nasal cavity, paranasal sinuses and nasopharynx. Diagnostic Histopathology of Tumors, 3rd ed.

[10] Kerr EH, Hameed O, Lewis JS Jr, Bartolucci AA, Wang D, Said-Al-Naief N (2012). Head and neck mucosal malignant melanoma: clinicopathologic correlation with contemporary review of prognostic indicators. Int J Surg Pathol.

[11] Letievant JC, Poupart M, Ambrun A, Colin C, Pignat JC (2016). Single-center retrospective series of fourteen patients with mucosal melanoma of the nasal cavity and paranasal sinuses. Eur Ann Otorhinolaryngol Head Neck Dis.

[12] Patrick RJ, Fenske NA, Messina JL (2007). Primary mucosal melanoma. J Am Acad Dermatol.

[13] Moreno MA, Roberts DB, Kupferman ME (2010). Mucosal melanoma of the nose and paranasal sinuses, a contemporary experience from the M. D. Anderson Cancer Center. Cancer.

[14] McLean N, Tighiouart M, Muller S (2008). Primary mucosal melanoma of the head and neck. Comparison of clinical presentation and histopathologic features of oral and sinonasal melanoma. Oral Oncol.

[15] Eisen MD, Yousem DM, Loevner LA, Thaler ER, Bilker WB, Goldberg AN (2000). Preoperative imaging to predict orbital invasion by tumor. Head Neck.

[16] Bockmühl U, Minovi A, Kratzsch B, Hendus J, Draf W (2005). Endonasal micro-endoscopic tumor surgery: state of the art [article in German]. Laryngorhinootologie.

[17] Won TB, Choi KY, Rhee CS (2015). Treatment outcomes of sinonasal malignant melanoma: a Korean multicenter study. Int Forum Allergy Rhinol.

[18] Lombardi D, Bottazzoli M, Turri-Zanoni M (2016). Sinonasal mucosal melanoma: A 12-year experience of 58 cases. Head Neck.

[19] Nicolai P, Battaglia P, Bignami M (2008). Endoscopic surgery for malignant tumors of the sinonasal tract and adjacent skull base: a 10-year experience. Am J Rhinol.

[20] Clifton N, Harrison L, Bradley PJ, Jones NS (2011). Malignant melanoma of nasal cavity and paranasal sinuses: report of 24 patients and literature review. J Laryngol Otol.

[21] Thompson AC, Morgan DA, Bradley PJ (1993). Malignant melanoma of the nasal cavity and paranasal sinuses. Clin Otolaryngol Allied Sci.

[22] Göde S, Turhal G, Tarhan C (2017). Primary sinonasal malignant melanoma: effect of clinical and histopathologic prognostic factors on survival. Balkan Med J.

[23] Gore MR, Zanation AM (2012). Survival in sinonasal melanoma: a meta-analysis. J Neurol Surg B Skull Base.

